# Acceptance of Telemedicine by Specialists and General Practitioners in Cardiology Care: Cross-Sectional Survey Study

**DOI:** 10.2196/49526

**Published:** 2024-02-20

**Authors:** Felix Muehlensiepen, Marie Josephine Hoffmann, Jonathan Nübel, Yury Ignatyev, Martin Heinze, Christian Butter, Anja Haase-Fielitz

**Affiliations:** 1 Brandenburg Medical School (MHB) and Faculty of Health Sciences (FGW) Brandenburg Neuruppin Germany; 2 Autonomie, Gérontologie, E-santé, Imagerie et Société (AGEIS) Université Grenoble Alpes Grenoble France; 3 Department of Cardiology, Heart Center Brandenburg Bernau & Faculty of Health Sciences (FGW) Brandenburg, Brandenburg Medical School (MHB) Neuruppin Germany; 4 Department for Psychiatry and Psychotherapy, University Hospital Immanuel Klinik Rüdersdorf Brandenburg Medical School (MHB) Rüdersdorf Germany; 5 Institute of Social Medicine and Health System Research, Otto von Guericke University Magdeburg Germany

**Keywords:** acceptance, adoption, cardiac, cardiology, cross sectional, health services research, heart, preference, survey, telecardiology, telehealth, telemedicine

## Abstract

**Background:**

In the coming years, telemedicine will play a key role in health care. Especially in rural areas with weak infrastructure, telemedicine could be crucial to providing adequate and personalized medical care.

**Objective:**

We investigated the acceptance and preferences of telemedicine among cardiologists, internists, and general practitioners. In addition, we aimed to identify knowledge, explore factors that influence the decision to adopt or reject this technology, and create starting points for demand-oriented further research.

**Methods:**

We conducted a web-based survey between May 2021 and February 2022. The 34-item questionnaire covered a wide range of questions regarding knowledge, acceptance, and use of telemedicine in cardiology care. Participants (cardiologists, internists, and general practitioners) were contacted through their professional email addresses, through a QR code published in a regional health journal, and through X (formerly known as Twitter). After exclusion of questionnaires with missed values, multidimensional scaling and k-means clustering were performed. Participants were divided into 3 clusters (C1, C2, and C3) based on their attitudes toward telecardiology. C1 uses telemedicine for personal health and clinical practice; C2 shows reluctance; C3 uses telemedicine mainly clinically.

**Results:**

We contacted 929 physicians. Of those 12.1% (112/929) completed the questionnaires. Participants were 56% male (54/97), 29% female (28/97), and 2% (2/97) diverse (median age 50 years). About 16% (18/112) of the respondents currently use telemedicine daily, 14.3% (16/112) 3-4 times a week, and 43% (48/112) did not use telemedicine at all. Overall, 35.1% (34/97) rated their knowledge of telemedicine as very good or good. Most of the respondents replied that telemedicine could support cardiology care in monitoring of blood pressure and electrocardiograms (57/97, 58.8%, both), consultation (57/97, 58.8%), and extending follow-up time (59/97, 60.8%). Reported barriers to implementation were mostly administration (26/97, 26.8%), inadequate reimbursement (25/97, 25.8%), and the purchase of technology equipment (23/97, 23.7%). Attitudes toward telemedicine in clinical practice were closely related to the number of patients being treated per annual quarter: C3 (median 1350, IQR 1000-1500) versus C1 (median 750, IQR 300-1200) and C2 (median 500, IQR 105-825). The differences between clinical caseloads of C1-C3 members were significant: C1 versus C2 (*P*=.03), C1 versus C3 (*P*=.02), and C2 versus C3 (*P*<.001). Most participants (87/112, 77.7%) would like to expand telemedicine approaches in the future. In the field of cardiology, the participants reported a high suitability of telemedicine. The willingness to train in telemedicine is high to very high for > 50% of the participants.

**Conclusions:**

Our results indicate generally moderate use but positive attitudes toward telemedicine among participating physicians with a higher clinical caseload. The lack of a structural framework seems to be a barrier to the effective implementation of telecardiology.

## Introduction

As the burden of patients with cardiovascular diseases (CVDs) is increasing [1], regions with an aging population, such as the German federal state of Brandenburg, are particularly affected. Demographic changes concern not only the patient population but also health care professionals (HCPs). The average age of physicians in the state of Brandenburg is 54.4 years. In the next 5-10 years, the number of physicians will decrease by one third, crucially impacting medical care in the sparsely populated states of Germany. There are already 683 inhabitants per contract physician in the state of Brandenburg [2]. At the same time, the digital transformation is radically changing health care delivery [3]. In rural areas, telemedicine could help to initiate treatment faster and might have a positive impact on quality of life [4]. In cardiology, telemedicine can be used in various ways, including remote patient monitoring, remote visits, and telecardiology consultations [5]. In patients with chronic heart failure, telemedical interventions were associated with optimized medical therapy, a significant reduction in hospital readmissions, and an improvement in quality of life [4,6,7]. As reported in other medical domains, telemedicine not only potentially extends the reach to underserved populations but also enhances opportunities to provide care within usual inpatient and outpatient settings [8]. Not to be underestimated for a future-proof and sustainable health care system, there are also first indications that telemedicine could make a significant contribution to reducing the carbon footprint of health care [9]. Yet, current data on the acceptance of telemedicine by HCPs in cardiology are lacking. Furthermore, the investigation of differences in telemedicine acceptance between urban and rural areas is a research priority. Thus, we aimed to assess the acceptance of telemedicine among cardiologists, internists, and general practitioners in Berlin and Brandenburg. In addition, we aimed to identify knowledge and explore factors that influence the decision to adopt or reject this technology. To create starting points for demand-oriented further research, we wanted to identify user types in the use of telemedicine.

## Methods

### Ethical Considerations

The study was approved by the local ethics committee of the Brandenburg Medical School (E-01-20210304). Data processing was based on the informed consent of the participants in the study. Participation in the study was not remunerated. Personal data were only collected from the participants to be able to process any requests in accordance with current law. These data were deleted after the end of the study. No other personal data were collected.

### Questionnaire and Procedure

The authors developed a web-based survey that was pretested and validated among cardiologists (n=5) and general practitioners (n=5). The final 34-item questionnaire covered a wide range of questions regarding knowledge, acceptance, and use of telemedicine in cardiology care. Physicians were asked to participate in the survey if they met the following inclusion criteria: (1) working in inpatient or outpatient cardiology care, (2) working in the states of Brandenburg or Berlin, and (3) providing informed consent. Consequently, physicians who did not meet these criteria were excluded. Furthermore, questionnaires were excluded if less than half of the questions were answered. Participants were contacted through their professional email addresses, through a QR code published in a regional health journal, and through X (formerly known as Twitter). Data were collected between May 28, 2021, and February 28, 2022, using the web-based survey application “LimeSurvey” [10], embedded in the domain [11].

### Data Analysis and Statistics

The statistical analysis was conducted in several steps. First, questionnaire data were analyzed using descriptive statistics, including quantities, percentages, median scores, and ranges for ordinal variables. An exploratory quantitative analysis was performed to identify factors determining telecardiology use. To create a data matrix for the analysis, qualitatively recorded participants’ attitudes toward telecardiology as reported in the survey were recoded from left to right according to the direction of the hypothesis of positive attitudes, for example, the response options in relation to the a priori hypothesis that “Would you like to use telemedicine more often?” which ranged from the positive to the negative scale. The answers “yes, totally,” “yes,” “no,” and “no at all” were coded as “5,” “4,” “2,” and “1,” respectively. The undefined “I don’t know” response option was coded with an intermediate value of “3.” Recoding was undertaken for the characteristics indicated by the age groups, the clinical location, gender, medical specialty, and type of practice. The interval-scaled variable “number of patients” (quarterly) was recoded. To ensure sample homogeneity, participants who did not use telemedicine were not included in the analysis.

To classify survey participants based on their attitudes toward telecardiology, multidimensional scaling (MDS) and subsequent k-means clustering were used. MDS aims to represent input proximities (typically dissimilarities) between objects by means of fitted distances in a low-dimensional space [12]. It therefore visualizes the level of dissimilarity among cases in a data set. Since we were interested in scaling the participants and not attitudes, the dissimilarity measure was applied across columns of the data matrix. Then, nonmetric MDS was applied to the dissimilarity matrix to obtain the coordinates of the sample in a representative low-dimensional space. The algorithm used for the MDS calculation was “*Scaling by Majorizing a Convex Function*” (*SMACOF* [13]). Starting with any initial configuration, the algorithm iteratively transforms proximities to estimated proximities (disparities) for calculating the configuration of the items in the context of the coordinates. This continues until the squared differences between the disparities and distances are minimized. These differences reflect a model’s (mis)fit, expressed by the Stress-1 index, which ideally has a value of 0; a value higher than 0.2 indicates a bad representation [14]. However, the larger the number of points (ie, participants in this study), the more difficult it is to map these into a low-dimensional space. This means that the Stress-1 index may become unacceptably high. For this reason, the Stress-1 index was criticized by Borg et al [15], who developed permutation tests for MDS solutions [16], whereas a significant test result (*P*<.05) allows to reject the H0, hypothesizing that the stress and, subsequently, the configuration are obtained from a random permutation of dissimilarities. As with other dimension-reducing methods, however, the final decision for an MDS solution should not be made based on these indices but on their interpretability [17].

To create a classification of participants based on their attitudes toward telecardiology, k-means clustering was applied to the mapped sample [18]. Clustering consists of grouping objects that are, in some sense, similar to each other. The k-means is a nonhierarchical clustering method commonly used in data mining [19]. The algorithm starts with a collection of s objects, where each object is a point in a q-dimensional space, and a given number of clusters, K, that is subjective and specified in advance by the user. However, to determine K, we also relied on the total Within Sum of Squares (WSS) index. The k-means groups the “s” objects into K≤s clusters to minimize the objective function given by the sum of distances between the points and the centers of their clusters. The k-means arrives at a solution in which objects within each cluster are as close to each other as possible and as far from objects in other clusters as possible. Finally, the chi-square test and the Kruskal-Wallis nonparametric test [20] (due to nonnormal data distribution), followed by the posthoc analysis using the Conover-Iman test with Holm’s correction [21], were applied to examine the difference in selected variables between each cluster. A value of *P*<.05 was considered statistically significant.

All data analyses and graphics were done within the R environment (R Core Team). To run MDS, *SMACOF* [13], *ggpubr* [22], *magrittr* [23], and *dplyr* [24] packages were used. The k-means clustering was done based on *stats* (version 3.6.2 [25]) and *factoextra* [26] packages. To run Conover Test with Holm’s correction, the package *connover.test* [27] was used.

## Results

### Overview

A total of 929 physicians were contacted, of whom 112 (12.1%) responded to the questionnaires. Of which, 15 (13.4%) participants were excluded from the analysis because less than half of the questions were answered.

### Sample Characteristics

The data for this survey were obtained from 97 physicians. Most participants—48.5% (47/97) were cardiologists, 15.5% (15/97) were internists, 12.4% (12/97) were general practitioners, and 23.7% (23/97) were not yet specialists at the time of the survey or their status was not reported at the time of the survey (Table 1). Most respondents, 56.7% (55/97), were aged between 40 and 59 years and worked in the state of Brandenburg (69/97 71%) in medium-sized cities (40/97, 41%) (Table 1). Additionally,, more than half of the participants were men (54/97, 55.7%). The ratio of respondents from the participants practicing inpatient and outpatient care was 48% versus 42% (10% with no response), with most participants in outpatient care working in a single practice. Position types, hospital characteristics according to the number of beds, and patients treated per physician per quarter are shown in Table 1.

**Table 1:** Demographic data and characteristics of the participants.

			Cardiologists (n=47), n (%)	Other disciplines (n=50), n (%)	Total (n=97), n (%)	
**Age (years)**						
		20-29	0 (0)	5 (10)	5 (5)	
		30-39	6 (13)	10 (20)	16 (16)	
		40-49	15 (32)	8 (16)	23 (24)	
		50-59	21 (45)	11 (22)	32 (33)	
		60-69	5 (10)	5 (10)	10 (10)	
		70-79	0 (0)	0 (0)	0 (0)	
		>80	0 (0)	2 (4)	2 (2)	
		Not stated	0 (0)	9 (18)	9 (10)	
**Sex**						
		Diverse	2 (4)	0 (0)	2 (2)	
		Female	14 (30)	14 (28)	28 (29)	
		Male	29 (62)	25 (50)	54 (56)	
		Not stated	2 (4)	11 (22)	13 (13)	
**Working area**						
		Outpatient sector	17(36)	24 (48)	41 (42)	
		Hospital	30(64)	17 (34)	47 (48)	
		Not stated	0 (0)	9 (18)	9 (10)	
**Medical practice types**						41 (100)
		Solo practice	6 (35)	12 (50)	18 (44)	
		Group practice	5 (29)	6 (25)	11 (27)	
		Employed physician practices	2 (12)	5 (21)	7 (17)	
		Outpatient clinic	2 (12)	0 (0)	2 (5)	
		Other	2 (12)	1 (4)	3 (7)	
**Position types**						47 (100)
		Resident	0 (0)	10 (58)	10 (21)	
		Medical specialist	5 (17)	1 (6)	6 (13)	
		Senior physician	15 (50)	3 (18)	18 (38)	
		Consultant	10 (33)	3 (18)	13 (28)	
**Hospital characteristics (number of beds)**						
	0-50		0 (0)	0 (0)	0 (0)	
	51-100		1 (3)	1 (6)	2 (4)	
	101-150		1 (3)	1 (6)	2 (4)	
	151-200		1 (3)	0 (0)	1 (2)	
	201-250		2 (7)	1 (6)	3 (6)	
	251-300		6 (20)	2 (12)	8 (17)	
	301-350		1 (3)	1 (6)	2 (4)	
	351-400		4 (14)	2 (12)	6 (13)	
	401-450		0 (0)	0 (0)	0 (0)	
	451-500		4 (14)	2 (12)	6 (13)	
	>500		10 (33)	7 (40)	17 (37)	
**Number of patients** **(per quarter)**						
	0-200		11 (23)	3 (13)	14 (15)	
	201-400		4 (9)	8 (33)	12 (12)	
	401-600		5 (11)	5 (21)	10 (10)	
	601-800		4 (9)	2 (8)	6 (6)	
	801-1000		8 (17)	6 (25)	14 (15)	
**Federal state**						
	Berlin		15 (32)	2 (4)	17 (17)	
	Brandenburg		31 (66)	38 (76)	69 (71)	
	Other		1 (2)	10 (20)	11 (12)	
**Location (number of inhabitants)**						
	Rural community (<5000)		0 (0)	8 (16)	8 (8)	
	Small city (5000-20,000)		7 (14)	6 (12)	13 (13)	
	Medium-sized city (20,000-100,000)		20 (43)	20 (40)	40 (41)	
	Big city (>100,000)		20 (43)	7 (14)	27 (28)	
	Not stated		0 (0)	9 (18)	9 (10)	

### Telemedicine: Knowledge and Use

Overall, 64.9% (63/97) of respondents rated their knowledge of telemedicine as satisfactory, poor, or very poor, whereas 35.1% (34/97) rated their knowledge as very good or good (Table 2). The frequency of current telemedicine use is shown in Table 2, with 16% (18/112) of the respondents currently using telemedicine daily, 14.3% (16/112) using it 3-4 times a week, and 43% (48/112) reporting no use at all. However, 45.4% (44/97) answered that they would like to use telemedicine more often in the future. Overall, 52.6% (51/97) of the physicians surveyed indicated that there were barriers to the use of telemedicine. The top 3 barriers to the implementation of telemedicine, according to respondents, were administration (26/97, 26.8%), inadequate reimbursement (25/97, 25.8%), and the purchase of technology equipment (23/97, 23.7%).

**Table 2:** Knowledge and use of telemedicine.

Question and responses			Frequency, n (%)
**How do you rate your own knowledge of telemedicine?**			
	Very good	9 (9)	
	Good	25 (26)	
	Satisfactory	41 (42)	
	Poor	18 (19)	
	Very poor	4 (4)	
**How often do you use telemedicine?**			
	Daily	16 (16)	
	3-4 times a week	14 (14)	
	3-4 times a month	14 (14)	
	3-4 times a quarter	13 (13)	
	Not at all	40 (43)	
**Would you like to use telemedicine more often?**			
	Yes, totally	25 (26)	
	Yes	19 (20)	
	I don’t know	8 (8)	
	No	4 (4)	
	Not at all	1 (1)	
	Not answered	40 (41)	
**Does anything prevent you from using telemedicine?**			
	Yes	20 (21)	
	Rather yes	31 (32)	
	I don’t know	15 (15)	
	Rather no	21 (22)	
	No	10 (10)	
**What prevents you from using telemedicine? (Multiple selections were possible.)**			
	Administration	26 (27)	
	Present insufficient reimbursement	25 (26)	
	Purchase of technology equipment	23 (24)	
	No reimbursement	17 (18)	
	Data security	13 (14)	
	Poor internet connection	12 (13)	
	Lack of data for patients benefits	10 (11)	

### Implementation of Telecardiology

Most of the respondents replied that telemedicine could support cardiology care in the monitoring of blood pressure and electrocardiograms (57/97, 58.8%, both), consultation (57/97, 58.8%), and extending follow-up time (59/97, 60.8%) (Table 3). When asked which communication partners they should exchange through telemedicine, 80.4% (78/97) responded “physician-to-patient,” 72.2% (70/97) responded “physician-to-physician,” and 51.5% (50/97) responded “physician-to-assistant or other participants” (multiple replies were possible). According to the respondents, the diseases or conditions that are particularly suitable for telemedicine care include “cardiac arrhythmias” (78/97, 80.4%), “monitoring of various diseases and conditions” (75/97, 77.3%), and “therapy” (74/97, 76.3%) (Table 3).

**Table 3:** Implementation of telemedicine in cardiology care.

Question and responses		Yes, n (%)	No, n (%)
**Which partners should establish communication through telemedicine? (Multiple selections were possible.)**			
	Physician-patient	78 (80)	19 (20)
	Physician-physician	70 (72)	27 (28)
	Physician-assistant	34 (35)	63 (65)
	Other participants and combinations	16 (17)	81 (83)
	No communication	4 (5)	93 (95)
**At which stages can telemedicine support cardiological care? (Multiple selections were possible.)**			
	Widen the time of follow-ups	59 (61)	38 (39)
	Consultation	57 (59)	40 (41)
	Blood pressure monitoring	57 (59)	40 (41)
	ECG^a^ monitoring	57 (59)	40 (41)
	Acute situations (eg, sending ECG to the hospital)	53 (55)	44 (45)
	Weight monitoring	49 (51)	48 (49)
	Complications	30 (31)	67 (69)
	At no stage	2 (2)	95 (98)
**Which cardiologic diseases could be monitored by telemedicine?**			
	Cardiac arrhythmias	78 (80)	19 (20)
	Monitoring of various diseases and conditions	75 (77)	22 (23)
	Therapy	74 (76)	23 (24)
	Myocardial infarction	73 (75)	24 (25)
	Hypertension	56 (58)	41 (42)
	No disease	2 (2)	95 (98)

^a^ECG: electrocardiogram.

### Telecardiology User Groups

**Figure 1:**
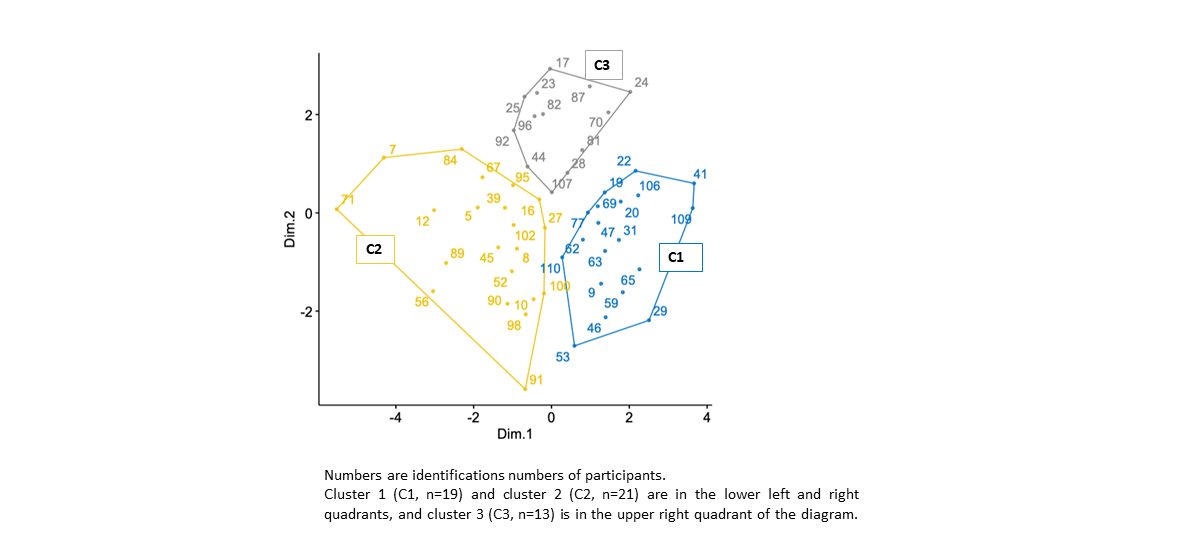
Segmentation of survey participants in a 2D group space. Numbers are identification numbers of participants. Cluster 1 (C1, n=19) and cluster 2 (C2, n=21) are in the lower left and right quadrants, and cluster 3 (C3, n=13) is in the upper right quadrant of the diagram. Telecardiology user groups were identified through cluster analysis of a web-based survey conducted between May 2021 and February 2022 to assess telemedicine knowledge, acceptance, and use. Analysis of 53 cases reveals 3 distinct clusters (C1, C2, and C3) based on use behavior. C1 uses telemedicine for personal health and clinical practice; C2 shows reluctance; and C3 uses telemedicine mainly clinically. Statistically significant differences were observed: C1 versus C2 (*P*=.03), C1 versus C3 (*P*=.02), and C2 versus C3 (*P*<.001).

The exclusion of the cases with missed values yielded a data matrix of 53 cases. Figure 1 shows a 2D MDS solution for the distribution of survey participants. The Stress-1 index was borderline (stress 0.20). However, the permutation test indicated a well-fitting model (*P*<.001). Considering the fact that the next step of the analysis involved more detailed clustering using k-means, the 2D solution was sufficient and more complex solutions were not examined. Determining the cluster numbers with the WSS index resulted in a 3-cluster solution. The application of this solution for the segmentation of the study participants using k-means showed that all 3 clusters were localized and ordered according to the axes of the MDS diagram. Cluster 1 (C1, n=19) and cluster 2 (C2, n=21) were in the lower left and right quadrants, and cluster 3 (C3, n=13) was in the upper right quadrant of the diagram. Table 4 shows that physicians assigned to group C1 used telemedicine privately to improve their personal health, yet not only in their clinical practice. Physicians in group C2 showed reluctant attitudes toward telemedicine. Members of group C3 use telemedicine for clinical activities and not for their personal health. Attitudes toward telemedicine in clinical activities were closely related to the number of patients being treated. This could be confirmed by a higher patients’ number per annual quarter (median 1350, IQR 1000-1500) treated by C3 members compared with the corresponding numbers indicated by members of C1 (median 750, IQR 300-1200) and C2 (median 500, IQR 105-825). These differences were statistically significant with C1 versus C2 (*P*=.03), C1 versus C3 (*P*=.02), and C2 versus C3 (*P*<.001). The comparison of other characteristics did not have any statistical significance.

**Table 4:** Comparison of attitudes toward telemedicine between members of 3 clusters.

Item	Cluster								K-W test^a^: *P* value			C-I test^b^				
	C1 (n=19)		C2 (n=21)		C3 (n=13)						C1 vs C2			C1 vs C3		C2 vs C3
	Mean (SD)	Median (IQR)	Mean (SD)	Median (IQR)	Mean (SD)	Median (IQR)				*P* value			*P* value		*P* value	
How do you rate your own knowledge of telemedicine?	3.53 (0.96)	4 (3-4)	3.48 (0.81)	3 (3-4)	4.00 (0.82)	4 (3-5)	.12^c^			.11			.14		.12	
How often do you use telemedicine?	3.53 (1.02)	4 (3-4)	3.33 (1.15)	3 (2-4)	4.38 (0.96)	5 (4-5)	.02			.31			.02		.0096	
Would you like to use telemedicine more often?	4.47 (0.77)	5 (4-5)	3.52 (1.03)	4 (3-4)	4.62 (0.87)	5 (5-5)	<.001			<.001			.21		<.001	
Does anything prevent you from using telemedicine? (reverse coding)	2.74 (1.41)	2 (2-3.5)	2.48 (1.17)	2 (2-3)	3.15 (1.28)	4 (2-4)	.22			.20			.17		.21	
How do you assess the need for relevant training on telemedicine among colleagues?	4.37 (0.60)	4 (4-5)	3.14 (0.85)	3 (3-4)	4.23 (0.60)	4 (4-5)	<.001			<.001			.09		<.001	
How do you assess the willingness of colleagues to undergo further training on the subject of telemedicine?	3.32 (1.06)	4 (2.5-4)	2.86 (0.85)	3 (2-3)	3.38 (0.96)	4 (3-4)	.21			.17			,20		.23	
How high is your own willingness to participate in training courses on telemedicine?	4.26 (0.93)	5 (4-5)	3.05 (0.86)	3 (3-4)	4.15 (0.69)	4 (4-5)	<.001			<.001			.08		<.001	
Would you be willing to (financially) invest in the application of telemedicine in your everyday care routine	3.79 (0.63)	4 (3-4)	3.00 (0.89)	3 (2-4)	3.92 (0.86)	4 (3-5)	.008			.007			.07		.009	
Do you use telemedicine applications (privately) for your own health?	4.32 (0.75)	4 (4-5)	2.10 (1.04)	2 (1-2)	1.69 (0.85)	2 (1-2)	<.001			<.001			<.001		.09	

^a^K-W test: Kruskal-Wallis-test; *P* values displayed.

^b^C-I test: Conover-Iman test with Holm correction; *P* values displayed.

^c^NS: Not significant.

## Discussion

Cardiologists, internists, and general practitioners consider the overall use of telecardiology to be acceptable; two-thirds of respondents would like to use telemedicine in their daily practice. However, most physicians rate their knowledge as “satisfactory” or worse, and less than a third were using telemedicine at the time of the survey. Barriers to telemedicine adoption, such as “limited knowledge,” “administrative burden,” “purchase of technology,” and “inadequate reimbursement,” were clearly identified by both specialists and generalists. Direct communication with patients is preferred to information exchange with colleagues. In the exploratory analysis, we found 3 potential telecardiology user groups, which differ regarding the number of patients treated per quarter: The more patients treated, the higher the telemedicine acceptance rate. The results provide information on how telemedicine can support cardiology care from the physicians’ perspective.

In 2021, a number of changes were introduced for telemedicine in Germany, such as the mandatory electronic patient record, an increasing number of prescriptible digital health applications, and video consultations for nonphysician therapists. The study TIM-HF II [7] has shown the world that telemedical interventional management can reduce mortality. Since January 2022, remote patient monitoring of patients with heart failure has been reimbursed by the statutory health insurance funds in Germany [28]. In addition to the reimbursement of costs, however, the expansion of telemedicine in the real world is the central topic of the implementation process that is now beginning. Yet, it is surprising that despite the successful study and the establishment of telemedicine infrastructure for cardiology care in the federal states of Brandenburg and Berlin, telemedicine acceptance among physicians in routine cardiology care is still heterogeneous. Considering further large-scale research activity on telehealth for prevention in hypertension care in this region [29], there seems to be a wide evidence-based practice gap for telemedicine in cardiology care [30]. Our results support this conclusion, as knowledge of telemedicine has been reported as low by the participants in this survey. Thus, we recommend high-quality training programs that reflect the multidimensionality of knowledge barriers by addressing the economic, organizational, and behavioral framework conditions of digital health implementation [31]. Furthermore, our results indicate that bureaucratic and infrastructural barriers hamper telemedicine implementation. These barriers were also identified in a similar study on telemedicine in rheumatology care, conducted before the COVID-19 outbreak [32]. This suggests the reported barriers to effective use of telemedicine have remained in Germany despite the pandemic and a massive digital health uptake globally.

COVID-19 has demonstrated the importance and acceptance of contactless approaches to medical care and, particularly, cardiology care [33-35]. Also, gold standard adherence measures in telemedical interventions need to be established so that study outcomes are more comparable [35]. As the survey was published in May 2021, it is not derivable from our data whether the willingness to use telecardiology has changed. Only a minority of the surveyed physicians currently use telemedicine, although two-thirds would like to implement telemedicine into their clinical routine.

It seems that the participants foreshadow and recognize a benefit that has already been described in the literature [7,36] but which does not yet seem measurable in its daily representants due to poor global implementation of telemedicine. Due to that lack of everyday application, it seems understandable that most physicians regard their knowledge of telemedicine as poor.

As physicians reported barriers to the use of telemedicine, the structural framework for effective implementation of telecardiology is not yet in place. Significant administrative burdens and inadequate reimbursement structures prevented the physicians surveyed from using telemedicine. The greatest barrier seems to be physicians' limited knowledge about “how to use telemedicine.” This underlines the need for clearly defined use cases for telemedicine in cardiology as well as the timely introduction of low-threshold training offers. Overall, this seems to reflect that the potential of telemedicine is not being fully reached. Further research should define use cases as well as specific interventions and evaluate the effects on patients’ outcomes and health and economic implications. Those seem particularly important because our data suggest that in the current health care system, only what is paid for is done. An increasingly aging society with an increasingly scarce resource of highly specialized doctors is catalyzing the need for enrollment in telecardiology under the aspects that PerplexityAI has already summarized: “telecardiology has the potential (…) to improve patient engagement and save time and money for patients and health care providers” [1]*.* Further research may therefore provide individualized patient- and clinician-adapted telemedicine options and triage mechanisms to select patients for either digital or analog consultations as appropriate. Based on our data, participants accept telemedicine and support its expansion if framework conditions such as reimbursement, the removal of existing usability barriers, and specialized training are optimized. The authors see this as a call to the health care system to create a framework for the use of telecardiology to optimize the use of an increasingly scarce resource with increasing demands and workloads. The provision of high-quality cardiology care using telemedicine will require urgent research, as well as the removal of existing barriers and training for specialists and generalists.

Due to the design of our questionnaire as a web-based survey, we assume a positive selection bias for physicians who are already interested in digitalization, telemedicine, or telecardiology. In addition to digital invitations to participate, participants were also recruited by an analog magazine report about the research project in the “*KV-intern*,” which was delivered to every physician registered with the Medical Association Brandenburg (Kassenärztliche Vereinigung Brandenburg). Either way, internet access and at least a certain level of digital expertise were required for participation. As the average age of the participants is comparable to the average age of physicians in Brandenburg [2], the group of participants surveyed nevertheless appears to be representative.

At this point, we have only explored the perspectives of physicians on telemedicine in cardiology. There is an urgent need to investigate the patients’ perspective on telemedicine implementation in cardiology care.

In summary, our results indicate low use but high acceptance among participating physicians. Although potential users report general willingness and the potential benefits of telemedicine, self-reported knowledge is limited. The lack of a structural framework seems to be a barrier to the effective implementation of telecardiology.

## Abbreviations

CVD: cardiovascular disease
HCP: health care professional
MDS: multidimensional scaling
SMACOF: Scaling by Majorizing a Convex Function
WSS: Within Sum of Squares

## Data Availability

The data sets generated during this study are available from the corresponding author on reasonable request.
